# Foreign Summary

**Published:** 1839-05-18

**Authors:** 


### FOREIGN SUMMARY.

*Paralysis of the first and second Branches of the Sensific Root of the Fifth pair of Nerves; Case of Goulding.—Anatomy of the Fifth pair of Nerves.—Application of it to Diagnosis of the Case.—Paralysis of the Portio Dura.—Paralysis of the third Branch of Fifth.—Paralysis of Fifth and Seventh combined.—Classification of Paralytic Affections of the Face.—Treatment and Recovery of Goulding.* By Dr. Corrigan, of Dublin.

Case.—Catherine Goulding, æt. 23, was admitted into the hospital 24th October, 1838. Six months before this date, she fell on her temple against an iron grate, and at the time felt a very acute pain in the part, with numbness in the left side of the head and face. The pain having continued, and her sight in the left eye having grown dim, she took some purgative medicine, and after this she continued pretty well until about two months before admission, when the pain of the temple returned, followed by almost total loss of vision in left eye. On admission, almost total loss of sight in left eye, with a very sluggish iris, a clear and a dilated pupil. She suffered from thirst, loss of appetite, and debility. Her bowels were confined; her tongue was white. The treatment adopted was leeching the temples, blisters to the back of the neck, and mercury pushed to active salivation. These measures greatly alleviated her symptoms, but on the 8th November there was a return of the pain in the temple, with dimness of vision, and followed on the succeeding days by tingling and numbness in the left side of the head, and extending down the face. A blister to the side of the head alleviated these symptoms, and the sight of the left eye continued to improve, but the numbness of face increased; and on the 10th December the following is the report of her state:—

She is slightly salivated (she had been using gr. iij. of pilula hydrargyri ter in die.) Over the left side of the scalp and in the ear there is undiminished sensibility, as also in all the portion of the cheek, which is below a line drawn from the angle of the mouth to the lobe of the ear. But in the left half of the forehead, the left eyebrow, around the left eye, and in the anterior part of the cheek, and in the left half of the nose within and without, and in the left half of the upper lip, there is total loss of sensation; so that in any of those parts the skin may be pricked with a needle without her being conscious of it. In the left half of the upper gum there is also total loss of sensation. In the lower gum the sensation is duller than natural. There is no loss of muscular power in the jaw or eyelid, nor any appearance of paralysis either when the face is at rest or when she speaks. In the left temple there was a circumscribed spot, which was very painful on pressure, and which, when pressed upon, gave her a shooting pain down the cheek. Over this spot she was leeched every second day. Her sight improved, the pupil became more active, but the other symptoms remained as before, with the addition that on the 21st December she complained of inability to move the jaw freely on the left side. There is, however, no want of power in the left eyelid. The repeated leeching has diminished the soreness of the temple and improved the sight very much, and she is now rubbing the scalp with tartarized antimony and mercurial ointment, and taking internally 10 grs. of hyd. c. magnesia ter in die.

This case is a valuable addition to our knowledge of the affections of the nerves of the face, which, until late years, was little better than a web of confusion. To Sir Charles Bell we owe the first clue that has led us out of the labyrinth; and the case before us, of Goulding, deserves a place among those to which we may refer as establishing the sureness of the foundation on which our opinions of the functions of the nerves of the face now rest.

To understand the nature of Goulding's case, it is necessary to call your attention to the anatomy of the fifth pair of nerves. There is in this case total loss of sensibility of the left side of the forehead, of the left side of the nose, of the left side of the palate, and of the gum of the left half of the upper-jaw, and of the upper-jaw, and of the cheek, as low as the angle of the mouth; while, below a line extending from the angle of the mouth to the lobe of the ear on the same cheek, the sensibility is perfectThere. is not the slightest loss of muscular power in any part of the cheek; she has full power over the eyelid, angle of the mouth, buccinator muscles, &c., whether in chewing, sneezing, laughing, &c.

The anatomy of the fifth pair of nerves will now explain to us this case, which is one of those beautiful instances in which anatomy, physiology, and pathology, mutually throw light on each other. The fifth pair of nerves consists, you will recollect, of two roots; one of the sensitive —the other the motor root. The sensitive root, after having formed upon it the Casserian ganglion within the skull, sends off from this ganglion three branches; the first the ophthalmic branch, which is distributed to the parts within the orbit, and which sends off the supra-orbital branches to the skin and integuments of the forehead, with a brush of smaller twigs which are distributed over the inner canthus of the eye and the root of the nose, while the nasal branch is spread over the alæ and tip of the nose. The second branch, the superior maxillary, given off from the Casserian ganglion, leaves the skull through the foramen rotundum, and sends along the infra-orbital canal, in the floor of the orbit—the infra-orbital branch, which passing out to the cheek through the infra-orbital foramen, is then distributed to the anterior part of the cheek to the ala nasi, and twigs of it descend as low as the external angle of the mouth; where they meet those coming up from the foramen mentale. Other twigs of this second branch, the superior maxillary, are distributed to the palate, the gum of the upper jaw, and the interior of the nose.

I have now to turn your attention to the third branch of this sensitive root of the fifth. This branch, setting out also from the Casserian ganglion, leaves the skull by the foramen ovale, in company with the motor root of the fifth pair. This motor root, which has lain in the skull behind the Casserian ganglion, has as yet formed no junction with any portion of the sensific root; but having passed out through the foramen ovale, in company with the third branch, it then, in the pterygoid fossa, becomes intimately interwoven with this third branch of the sensific root, and the compound nerve, thus formed, is the inferior maxillary nerve. It is obvious that, according to this account of the anatomy of the fifth pair of nerves, the ophthalmic and superior maxillary nerves being given off by the sensific root of the fifth before any connection has as yet taken place between the sensific root and the motor root, the ophthalmic and superior maxillary nerves must be merely sensific nerves; and that in the event of disease producing paralysis of these nerves, the effect on the part supplied by them ought to be loss of sensibility alone; and that, as these nerves cannot confer motive power, muscular action should not be disturbed by paralysis of them; and thus exactly we have it in the case before us. There is loss of sensibility in all the parts of the face and interior of the mouth supplied by those branches, but no loss of muscular power. Thus anatomy and physiology explain to us those symptoms which otherwise would be inexplicable; and again, pathology, more beautifully than a thousand experiments, confirms the accuracy of our anatomical observations, and the truth of our physiology of this nerve.

Sensation, we have seen, is perfect along the lower jaw below a line drawn from the angle of the mouth towards the lobe of the ear. The parts below this line, the skin of the cheek, the chin, and the gum of the lower jaw, are supplied by the third branch of the fifth, namely, the inferior maxillary nerve. Now, does the preservation of sensibility in the lower part of the cheek, while it is lost in the upper part—or, in other words, does the continuance of function in the third branch of the sensitive root of the fifth, while it is lost in the first and second branch, lead to any practical result in diagnosis and prognosis? With the light of anatomy it does, and to a very important one. It tells us that the disease which has deprived the first and second branches of the fifth of their function of sensibility is not disease of the brain, nor, probably, has it its seat within the cavity of the skull; for if the diseased action attacked the nerve previously to its forming the Casserian ganglion from which all three branches take their origin, then it would be nearly impossible that the function of the third or inferior maxillary branch should not have been equally destroyed with the functions of the other two. Were the disease within the skull, it is also most probable that the motor root of the fifth, which in part of its course lies in very close relation to the sensific root, should suffer equally. But as the motor trunk and the third branch of the sensific trunk are not injured, we are justified in concluding that the cause of the paralysis of sensibility of the first and second branches has its seat external to the cavity of the skull, and the diagnosis thus made leads of course to the more favourable prognosis. Thus, anatomy and physiology here lead us both to diagnosis and prognosis. With this instance before you to show you the value of the observation, let me entreat of you never to lay aside anatomy or physiology in studying practical medicine. How different now is our interest in this case, and how superior our knowledge of it, when we have thus taught ourselves that even in disease the symptoms which present themselves are not the result of mere chance, but are in strict accordance with the laws of healthy action! If there were no other results from this analysis of the symptoms of this case than the attractive investigation of some of the functions of the nervous system and the conformation of our physiological knowledge, this alone should make us study the symptoms with enthusiastic interest; but when we find that on this analysis depends our knowledge of the nature of the disease, its study then becomes a duty. There is, I am sorry to say, a growing tendency to substitute what is called observation at the bed side for anatomical and physiological investigation of structure and symptoms. Such a doctrine is very acceptable to the ignorant empiric and the indolent student; but to expect to attain sound knowledge by such a course, would be as reasonable as it would be in a mechanist to expect that he could ever attain a knowledge of the derangements of a machine, without bringing to his assistance a knowledge of its structure and its powers.

If we now turn our attention to the inferior maxillary nerve, the third branch of the sixth, and to the portio dura of the seventh, we shall find in the physiology of these nerves an explanation of some curious forms of nervous affections of the face, and to which we find almost nothing analogous in any other part of the body.

The first is that in which the seventh or portio dura alone is paralyzed.

I saw a lady some time since, whose features were undisturbed and free from distortion when at rest, but when she laughed or smiled, the muscles on the right side alone acted, while the left side of the face remained perfectly passive, thus giving to the countenance the hideous expression of half of a living and of a dead face being joined together. In such a case as this the paralysis is confined to the seventh nerve, the motor branch of the fifth being unaffected. While the face is at rest the motor branches of the fifth at each side neutralize each other, and there is no distortion; but on the seventh or portio dura being called into action as a nerve of expression, the nerve of one side alone acts, and thus this singular form of paralysis is produced.

In contrast with this may be placed a case of paralysis of the third branch of the fifth, the inferior maxillary nerve. The case is from Sir C. Bell's work on the Nervous System.

A man affected with hemiplegia was paralytic of the right side of the face, which was also insensible on being pricked with a needle.

When at rest, the right side of the mouth and the right cheek hung down, and the saliva constantly flowed from it. But when he was made to sneeze, to laugh, or to whistle, the distortion disappeared, and both sides of the face acted equally. Thus these cases are contrasted with one another. In paralysis of the portio dura alone, *there is no paralysis when the muscles of the face are at rest*, but it becomes most disagreeably evident by distortion on giving expression to the face. *In paralysis of the third branch of the fifth there is paralysis of the face when at rest;* but when a respiratory expression calls the seventh into action, the paralysis for the time being disappears.

The most common form of paralysis of the face is when both the third branch of the fifth and the portio dura are simultaneously affected so that there is paralysis both when the features of the face are at rest, and in the expression of laughing, sneezing, &c. Such is the following case:—

Michael Keefe, admitted into this hospital December 3. About ten days before, he felt his upper lip swelled, and next day perceived that, in attempting to masticate, he could not turn the morsel in the right side of his mouth. On admission his mouth was drawn to the left side; he was unable to close the eyelid of the right eye; he could not whistle, and when he laughed the left side of the face alone acted. In this case both the seventh and the third branch of the fifth of the right side were paralysed, for there was in the case a combination of the symptoms of the two former cases. There was inability to masticate, and there was distortion of the face when at rest, dependent on paralysis of the third branch of the fifth; and there was also distortion of the face in expression, indicating paralysis of the portio dura.

We have thus the following form of local paralysis of the face:—

1st. Paralysis of sensibility, as in the case now in hospital, the muscular power being unaffected—dependent on disease of first and second branch of the sensific root of the fifth pair of nerves.

2d. Paralysis, not visible when the features are at rest, but most strongly marked when any respiratory expression, such as sneezing, laughing, &c. is attempted—dependent on disease of portio dura.

3d. Paralysis, persistent when the face is at rest, but temporarily suspended when any respiratory expression is attempted—dependent on disease of the third, or compound motor sensific branch of the fifth.

4th. Paralysis of the face both at rest and in motion, in which both the portio dura and third branch of the fifth are implicated.

For the experiments and some of the cases on which I have grounded these observations, I must refer you to Sir Charles Bell's work on the Nerves. There is, however, in some parts of that work an obscurity, and occasionally an apparent contradiction, which may render its perusal difficult. You will obviate the difficulty by consulting Dr. O'Beirne's analytical correction of Sir Charles Bell's work. It is published as an appendix to Dr. O'Beirne's work on Defæcation, and it will well repay you for a perusal. To return to our case in hospital. I have already given my reasons, founded on the immunity of the inferior maxillary nerve, for thinking that in this case the disease is not within the cavity of the cranium, or at least between the Casserian ganglion and the origin of the nerve. It is probable that the injury which this patient received produced periostitis of a subacute or chronic form, and that this, creeping along the temporal surface of the sphenoid bone, has also spread through the sphenoid-maxillary fissure to the lining membrane of the orbit, and has then involved the first and second branches of the fifth. This view is strengthened by the circumstance of their being still a small space in the upper part of the temporal hollow which is painful under pressure. I shall therefore, for some time longer, continue the same line of treatment under which he at present is, viz. repeated local blood-letting and blistering, and the exhibition of a mild course of mercury. The head symptoms, the vertigo, &c. have ceased, the motion of the pupil has become natural, and the sight is much improved, so that we have to contend with only the local tenderness in the temple, and the loss of sensibility of the first and second branches of the fifth.

P. S.—Since the above was delivered, numbness, accompanied with loss of power, began to extend to the lower jaw, showing that the diseased action was beginning to implicate the third, or compound branch of the fifth. The whole head was then rubbed over with antimoniated mercurial ointment and iodine; the amendment was rapid, and she soon after left the hospital with scarcely a trace of the disease.—*Lon. Med. Gazette.*

---

*On Iodine in Enlargement of the Tonsils.* By Sir B. C. Brodie.—Some short time since there was a boy in the hospital, under the care of Sir B. Brodie, with enlarged tonsils, which were touched every day with a camel's hair painting-brush, dipped in the tincture of iodine. Sir B. Brodie remarked, that in some cases of the kind he had known children get thin from the employment of this powerful medicine, in the above form. He supposed that such an effect might arise from a small quantity of the tincture being swallowed (which could hardly be prevented,) or from its being absorbed from the mucous membrane covering the tonsil. Sir B. Brodie further observed, that iodine was a very dangerous medicine, and required much caution in its administration, and that he could bring forward at least four instances of persons dying from taking too large doses of iodine. One of these cases was a gentleman who laboured under some irrritation of the prostate gland, accompanied with some urethral discharge, which, under his (Sir B. Brodie's) directions, was always relieved by the temporary use of the catheter, and the decoction of the pareira brava. He, however, got tired of this plan of treatment, and some time afterwards he (Sir B. Brodie) was called to him, and found him in a dreadful state, with great irritation of the prostate gland and bladder, and with a thick, ropy, mucous discharge. He eventually died, and on looking over his prescriptions, he found that he had been taking large doses of iodine and hydriodate of potash. He (Sir B. Brodie) was really afraid to say how much these medicines had been prescribed for him by a homœopathic doctor.* What added to the positive proof that all this dreadful train of symptoms was caused by the iodine was, that not one of them showed themselves before he took it.—*Lancet.*

* So we understood Sir B. Brodie to say, although the large quantity spoken of would appear to be somewhat contrary to the usual "dwarf" doses generally prescribed by this singular class of practitioners.

---

*On Disease of the Kidneys.* By Sir B. C. Brodie.—I have frequently had occasion to draw your attention to cases of diseased kidney, simulating diseased bladder; and the diagnostic marks by which you may distinguish the one from the other, are these. In disease of the kidney the symptoms generally consist of a frequent desire to void the urine, pain along the urethra, and pain referred to the pubes and neck of the bladder after making water; the urine is in general acid, and almost always albuminous. I had a case a short time since which strikingly illustrated the truth of this diagnosis. A gentleman came to me with all the above symptoms; I tested his urine, both by nitric acid and by heat, and it proved to be always albuminous. I found, upon further inquiry, that he had voided a small calculus when he was a child, and that he had been subject to irregular attacks of fever, one of which had preceded the symptoms for which he consulted me. He died eventually of a disease which I do not think I ever met with before, *viz.*, ulceration of the coats of the gall-bladder from the pressure of a gall-stone, and consequent effusion of bile into the peritoneal cavity. I had the opportunity of examining this case after death; I found the urinary bladder perfectly healthy, but the kidneys were diseased. One of them was soft and pulpy, and quite degenerated in structure; in the other I found two calculi, closely invested by surrounding membrane, and a large deposit of urine above them. It is not always that you have the opportunity of examining these cases before disease in the bladder is set up, which it always is eventually if the disease remains unchecked. These cases are sometimes very puzzling, for it is not in every case that you have, that medicine is of any avail; for what can you do with your medicines when a patient has a calculus imbedded in the kidney? Why, you can do nothing. In cases where this does not occur, you will find the patient derive much benefit from the uva ursi, or the wild carrot-seed tea. With reference to a case of diseased kidney that was some short time since in the hospital, Sir Benjamin Brodie observed that such disease would, if it ran its course, bring on diseased bladder, diseased prostate gland, calculi in the bladder, and diseased testis, and the converse might be said of many of these latter diseases bringing on irritation in the kidney. Sometimes the secondary disease was the first one which attracted the notice of the surgeon, and was in many instances produced at first by sympathy only, which in the end degenerated into real disease, in which way only could be explained the identity of affection between the kidney and bladder in these cases. Sir B. Brodie related the case of a lady who had disease of the bladder from the impaction of a mulberry calculus in the kidney.

Some time since a boy was admitted into the hospital under Sir B. Brodie's care. He laboured under severe pain in the groin, with inability to pass his water. These symptoms were soon relieved by the use of appropriate remedies. At times he passed several small calculi by the urethra, which, on being carefully analyzed by Dr. Prout, proved to be phosphatic in their species and composition. The patient had also some pain in the region of the kidney, but no particular irritability of the bladder manifested itself, except when any difficulty occurred in passing his urine, from portions of phosphatic calculi stopping up the passage of the urethra.

He died about thirteen months after his admission, and on a post-mortem examination being made, the following appearances presented themselves:—

The general external appearance of the body was œdematous, and there was some redness over the upper part of the thighs, from urinary excoriation. On opening the abdomen it was found ro be ascitic; the liver was hypertrophied, and densely gorged with blood, but its internal structure revealed no organic change. The general intestinal superfices was redder than natural; the appearance of the stomach presented nothing abnormal. On examining the kidneys (the pathological appearance of which it was expected would reveal the cause of death,) the left one was found to be much larger than natural, and its general structure engorged with blood. Its external surface was soft, spongy, and easily friable, and dotted with numerous small cysts, containing serum. On cutting into it, it was found to be highly injected, and two minute portions of calculi were discovered, each weighing, we should presume, at least half a grain; these were supposed by Sir B. Brodie to consist of phosphate of lime, and were taken to Dr. Prout for analyzation. The right kidney was much diminished in size, and softened in texture, but presented no other unusual abnormal appearances worthy of being recorded. The canal of each ureter was enlarged to double its natural character; the bladder was contracted in size, and its external surface was rugose and pouchy.

The chest, the lungs, and bronchial apparatus were found in a healthy state. The cavity of the pericardium was much distended with fluid; and on laying open the heart, the walls of the left side were found to be enormously thickened and extensively hypertrophied, but no disease of the valvular apparatus could be discovered.

Sir Benjamin Brodie remarked, that to account for the thickened state of the walls of the heart there must have been some obstruction to the circulation somewhere; where that obstruction was, however, this dissection did not reveal. Some cases of aneurysm of the aorta, combined with diseased kidney, were detailed by Sir Benjamin, but as they bore no similarity to the present case, inasmuch as aneurysm did not exist, we have not recorded any notes of them.—*Ibid.*

---

*Ulcerative absorption of the coats of the bladder, from the pressure of a fibrous tumour of the Uterus.* Dr. T. Thomson exhibited a tumour connected with the uterus, of which he related the following particulars:—He was sent for, on Sunday evening, to see a dispensary patient, who had not made any water for five days. He found the woman to be 40 years of age, and in a sinking state; the pulse was not to be detected, and the heart's action nearly ceased. The abdomen was much distended, and, on percussion, indicated a very full state of the bladder; the upper part of the abdomen, on one side, was tympanitic; the sphincter ani was relaxed, and a quantity of fluid resembling urine flowing from the rectum, and there were spots of blood on her dress. He was at first inclined to think that the bladder had burst into the rectum. She had turned round in bed just before, and he thought the motion might have ruptured the distended viscus. The woman died in half an hour. The body was examined on Thursday. The abdomen was still very prominent, half way between the umbilicus and the pubis a hard tumour could be felt. On opening the abdomen the bladder was found much distended, and there was a quantity of urine in the abdominal cavity. Four quarts of fluid were removed from the bladder. It was then found that the pelvis was almost entirely occupied by a large fibrous tumour and two or three smaller ones, which had grown from the fundus of the uterus; one of the smaller growths had pressed upon the posterior wall of the bladder, and produced ulcerative absorption, the tumour entirely filling up the cavity thus made, and preventing the escape of the urine. The anterior parietes of the bladder were pressed against the back of the symphysis pubis. The bladder was otherwise entirely healthy; there was very little peritoneal inflammation. The left ureter was much distended; the right was of the natural calibre; the kidneys were healthy. There was a collection of fæces above the rectum. Regarding the previous history of the case, little that was satisfactory could be ascertained. It appeared that she had complained of the presence of a lump on the right side of the abdomen, but it was neither painful nor tender. On one occasion (twelve months since) she had been under the care of a dispensary physician for a week or two, for difficulty in making water, which symptom was treated by spirits of nitre, and relieved. She had occasionally suffered from a pain extending down the right thigh, and had for some months made very small quantities of urine. The catamenia were regular. Dr. Thomson offered the following explanation of the case and its fatal result. The smaller tumour had pressed against the posterior walls of the bladder, and produced ulcerative absorption, the ulcer being of an irregular shape, the irregularity being accounted for by the various degrees of distension of the bladder at different times, according to the quantity of urine it contained. The tumour so entirely filled up the cavity produced by the ulcerative process as to prevent any escape of the urine, and this escape did not occur until a short time previous to death, when the patient, changing her position in bed, altered the situation of the tumour, and egress was given to the urine. The immediate cause of death was the shock produced upon the system by the escape of the urine into the peritoneal cavity, death being produced before inflammation to any extent could set in. He was inclined to the latter opinion rather than to the idea of her having perished from retention of urine, none of the usual symptoms indicative of the evil effects of the latter state being present, the only sign of head affection being a slight vomiting. The tumour was the usual fibrous tumour of the uterus, and had no trace of a malignant character about it. He had been able to trace vessels in its substance, proceeding from the centre to the circumference, a fact worthy of attention, as many high authorities had doubted whether this description of tumour was supplied with vessels. The case was curious from the fact of a tumour, of a simple fibrous structure, producing ulcerative absorption.

A discussion, embracing a variety of points connected with the tumour, took place. Had the presence of the disease been accurately ascertained before death, could any remedial means have been employed with a good effect? It was argued, on one side, that the position of the tumour might have been altered, so as to prevent its pressing always on the same portion of the bladder, and that the introduction of the catheter would have assisted, by keeping the bladder free from distension, in taking off the pressure; while, in opposition to this, it was contended that, at all events, in the latter stages of the disease, the drawing off the urine would have disturbed the position of the plug, and the urine would have escaped into the peritoneum, even before it had done. Regarding the mode in which the tumour had acted on the bladder, it was argued by one speaker that it could not have produced ulcerative absorption through the serous structure without marks of ulceration being present, which was not, however, the case; that the appearances observed around the hole in the bladder were rather those of the viscus having given way from distension, than from the process of ulceration, the peritoneal covering being lacerated to a greater extent than the other tissues. In opposition to this it was contended that absorption from pressure simply might have occurred, without any inflammatory appearances being detected, and that the peritoneal coat would necessarily be more extensively acted upon than the other tunics, from the fact of the pressure having been exerted upon it for a longer period.—*Ibid.*

---

*Clinical Lecture.* By Mr. Arnott. 1. *Foreign Bodies under the Upper Eyelid.* 2. *Deep-seated yellow Opacity in the Eye, not dependent on Medullary Fungus.* 3. *Penetrating Ulcer of the Cornea—Hernia Corneæ.* 4. *Blepharospasmus.*—After some remarks on the principal points to be attended to in the examination of diseased eyes, with a view to diagnosis, Mr. Arnott observed, that of injuries to this organ the most common are those produced by the access of foreign bodies to it. Most of these act mechanically; some by their continued presence causing irritation and inflammation; others, according to their form or the impulse given to them, producing a wound followed by the same results, or destroying the eye at once as an organ of vision.

A small particle of any substance getting into the eye, as is said, instead of being washed away by the copious flow of tears which immediately ensues, or being got rid of by the almost involuntary efforts of the patient for its removal, sometimes remains; and it is important to know where and how to look for it.

In examining an eye in which a foreign body is alleged to be, you first run over that part of the organ naturally exposed; look at the edge of the lids, to ascertain that it is not resting there—to the internal angle, where it is sometimes entangled—to the cornea, where sharp bodies are frequently imbedded—and to the exposed part of the conjunctiva. Not finding it, gently pull down and evert the lower lid, so as to expose its inner surface, and cause the patient at the same time to direct the globe upwards, so as to bring into view the whole of that gutter formed by the reflection of the conjunctiva from the lower lid to the globe. Failing to discover the foreign body here, you then evert the upper lid; and this is easiest affected by taking hold of the ciliæ with the finger and thumb of the left hand, and having placed a probe on the outer surface of the lid, just behind the posterior edge of the tarsal cartilage, you turn the lid over on the probe, so as to evert and expose its inner surface.

The inner surface of the upper lid is a very common seat of foreign bodies. A man of the name of Carne was admitted on the 26th of Feb., into Hertford ward, with iritis, the symptoms of which, under the ordinary treatment, soon subsided. Some days after the redness had disappeared, it was observed to have recurred; but this was now seated exclusively in the conjunctiva, and the patient was questioned as to the cause, whether he had been out, &c. &c. He denied having been out, adding, however, that for a day or two he had felt as if something was in the eye. There was nothing observable in the exposed part of the organ, nor on the inner surface of the lower lid; but on everting the upper, there was percieved adhering to its concave surface a minute black particle of matter, which required the application of the edge of the flat end of the probe to effect its displacement. The sensation of which the man complained was now gone, and the redness of the eye soon disappeared.

About a fortnight ago a surgeon in the army came to my house, complaining of great pain in the eye, which was red, watering, and which he could not open voluntarily, arising from something having got into it two days previously. He had tried to remove it, and having had the pipe of a small syringe placed under the upper lid (to which situation he referred it,) a stream of water had been thrown under the lid, in expectation of dislodging it, but ineffectually. On everting the lid, there was seated a small black speck on its inner surface, which being removed, the pain ceased, and the other symptoms followed.

A minute particle thus placed may remain for some time, so as to escape detecton. Last September a lady felt something get into her eye as she was walking in the street. Her surgeon, on repeated examinations, could discover nothing; and as, notwithstanding the use of lotions, &c., the sensation still continued, and the eye was red and weak, at the end of eight days I was requested to see her. On everting the upper lid there stuck on its concave surface a small black speck, which being displaced, its effects quickly ceased.

If it is not discovered in the situation we have been just alluding to, and the sensation of it is still present, then it may be entangled in that part of the conjunctiva which is reflected from the upper lid to the globe, and as this gutter cannot be brought into view, we sweep it by means of a camel's-hair pencil, dipped in oil, passing this under the lid to its whole depth, from the inner to the outer angle of the eye.

For removing substances partially imbedded in the inner surface of the upper lid, or in the cornea, the instrument I show you is useful; it is merely a thin piece of silver, the size of a cataract needle, but flattened like a minute scapula, having neither a sharp point or cutting edge, the edge being sufficiently thin to catch the projecting but partially imbedded foreign body, and yet not to wound the conjunctiva or cornea.

The substances impacted in the cornea are usually of metal, but any thing equally sharp will penetrate. A tradesman in Bond street brought me his son, who, two days before, felt something get into his eye, where it still remained. I detected it in the cornea, removed it with this instrument, and receiving it on the nail, it appeared to me to be a bit of black sealing wax, which the lad confirmed by stating that he was breaking a stick of it at the time he first experienced the sensation.

In connexion with the effects of injuries of the eye, the case of John Body, who was admitted on the 5th of December, presented some very interesting appearances. This lad had an affection of the left eye, the conjunctiva and sclerotica of which were red; the cornea, anterior chamber, and pupil clear, the latter of its ordinary size; the iris of its natural colour, but uninfluenced by the action of light, *i. e.* motionless. But the peculiarity of the case consisted in the existence at the bottom of the eye, deep behind the plane of the lens (probably posterior to the vitreous humour,) of a yellow substance, over the surface of which a number of the most delicate red vessels could be seen running, and presenting a very beautiful appearance. The vision of the eye was wholly lost.

Here were symptoms exactly corresponding to those presented by medullary sarcoma of the eye in its early stage; and most persons witnessing them for the first time, and ignorant of the history of the case, would have attributed them to that dreadful, and in the eye, utterly hopeless disorder. But though Body was admitted on the 5th of December, this was a readmission. He had originally come into the hospital three months and a half previously, viz. on the 21st of August, and under the following circumstances:—

A week previously a stick had been thrust into his left eye, and the sight immediately destroyed. The conjunctiva and sclerotica were now crowded with red vessels, with chemosis of the former membrane. The cornea was transparent, but a bluish turbidity filled the anterior chamber, so as considerably to obscure the pupil and iris. The former, however, appeared to be of its natural size, but the condition of the latter could not be satisfactorily made out. At the outer part of the globe there was a wound of the sclerotica just at its juncture with the cornea, which was also in a very trifling degree involved.

With this condition of parts, and total loss of vision, there could be no question as to the prognosis; it was hopeless as regarded the recovery of sight. But still the case was a proper one for treatment—active treatment, too—it being a most important object to save the form of the eye, although it is destroyed as an organ of vision. Here was an eye affected with violent inflammation in consequence of injury: if the inflammation had been allowed to run on unchecked, it might have terminated in suppuration and destruction; that is, bursting and collapse of the globe, and consequent falling in of the lids. Now, a sightless eye, if of its natural form and size, is much less a blemish; and hence, with a view of preserving this in Body, leeches were repeatedly applied, purgatives given, and the other parts of the antiphlogistic treatment adopted, followed by the exhibition of calomel in repeated doses, and the application of belladonna, in the hope of checking the progres of iritis, should it have existed, or of deeper-seated disorganizing inflammation.

Three days after admission the bluish tubidity in the anterior chamber had materially lessened; by the fifth, had entirely disappeared; and now there was observed deep behind the pupil, a greenish yellow colour, which by the eighth day had become uniformly saturated and yellow. From the first, it was evidently not seated in the lens, which had either disappeared by absorption in consequence of rupture of the capsule, of which the bluish turbidity in the anterior chamber had in the first instance given some suspicion, or it remained and was perfectly clear and transparent. Now, it was as certainly seen not to be seated in the posterior capsule, but deeper than this, possibly in the vitreous humour, but probably posterior to it. A small quantity of pus or lymph collected under the conjunctiva, at the part corresponding to the wound in the sclerotica, and evidently having exuded from within this coat. On being pricked with a cataract needle, a mere drop oozed out; this re-collected, but afterwards became less prominent, and gradually subsided.

On the sixteenth day after admission, the appearances as regarded the colour at the bottom of the eye were the same, but there was now for the first time observed several minute vessels running apparently over the substance on which the yellow colour depended, and directing their course from the periphery of the globe towards its axis; these increased in number, so as speedily to form quite a zone all round, apparently in a situation corresponding to the level of that of the posterior part of the ciliary body, or the anterior part of the vitreous humour, deeper than which the yellow substance consequently must have been. The redness of the conjunctiva and the sclerotica had now somewhat abated; the cornea remained clear; the pupil of its ordinary size; and the iris of its natural texture, but motionless.

The condition of the eye becoming stationary, and the lad anxious to get home, he was now made an out-patient, occasionally showing himself, until his readmission in December, when little or no change had taken place; the sole treatment in the interval having been irregularly taken doses of hyd. c. creta.

The history of this case, then, shows that the yellow appearance at the bottom of the eye, which was at one time presumed to be diagnostic of medullary disease, is not actually so; that this appearance may originate in inflammation, and in the deposition probably of lymph, in the situation usually occupied by the medullary growth. In this respect it confirms the observation of Messrs. Travers, Lawrence, and others, who have shown that this deposition may not only originate in inflammation arising from injury, but in the same process taking place deep in the eye, unconnected with injury.

The subsequent progress of our case corresponds with one related by Mr. Travers, of a young lady wounded in the same situation as Body. In a short time after his readmission, what I had for some time prepared you to expect took place—the globe diminished in volume; the cornea became less, the pupil contracted; in short, atrophy of the organ commenced, and was proceeding when he left the hospital.

The next case worthy of remark is that of Mary Falkner, æt. 25, who was admitted on the 5th February, on account of corneitis. The cornea of the left eye was the seat of an ulcer, occupying about one-fourth of its extent at the lower part. The ulcer was deep—had an irregular ragged appearance and yellow colour, as if the part had been subjected to maceration, and infiltrated with pus. There was very considerable redness of the sclerotic coat and conjunctiva, great pain, intolerance of light, and epiphora.

This patient had been confined in the Westminster Lying-in Hospital about a month previously, and shortly afterwards had been attacked with this affection in her eye, which, notwithstanding the treatment adopted, had become aggravated.

She was put upon low diet, purged, leeched repeatedly, and calomel in repeated doses was given, and belladonna applied round the eye, in case the iris was inflamed, which could not at first be satisfactorily determined. She complained much of an augmentation of pain at night, to obviate which, opiate frictions of the forehead were used.

On the fourth night after her admission, she had a very severe attack, followed by a sudden escape of watery fluid, and a sensation as if something had given way. The following morning I found that this had arisen from the ulcer having penetrated the anterior chamber, which was abolished, the iris being every where in contact with the cornea; but from this time the inflammation began to subside; the ulcerated surface lost its yellow appearance, and became clear and transparent. The previous treatment was persevered in, with the addition of a seton in the temple. On the fifth day after the ulcer had perforated the cornea, it was observed that the anterior chamber was re-established, and that there projected through the opening in the cornea a small transparent vesicle, constituting what is termed a hernia of the membrane of the aqueous humour, or keratocele, a condition dependent on the opening of this membrane having closed previous to that in the proper substance of the cornea, and being distended and protruded through the aperture in the latter, which takes a longer time to heal.

In the treatment of ulcer of the cornea, acute in its progress, as in the present case, the most efficient means are the antiphlogistic treatment, and issues in the temple. Calomel was given here, but this was done chiefly because it was dreaded that iritis also existed, for I do not attach so much value to its employment in ulceration of the cornea as to that of the ordinary depletory treatment. It has frequently been recommended that the nitrate of silver should be applied in substance or in strong solution to the ulcer; but you will hesitate to resort to this in cases like the present, where the ulcer was so manifestly the consequence of acute inflammation, and not the cause of that under which the eye laboured.

When hernia of the cornea exists, how is it to be treated? We have been advised to snip it off, or to touch it with nitrate of silver. I recommend you to do neither; as the ulcer heals it will disappear, though no doubt the ulcer of the cornea may, like ulcers elsewhere, exist for a considerable time. You saw how long this hernia was present in the case of Falkner, (upwards of a fortnight,) but that as the ulcer filled up and closed around it, it disappeared. But I have known it to persist for months. I was once consulted by an artist, in whom, according to the statement of the very intelligent surgeon who accompanied him, this affection had existed for four months. This gentleman had been subject every winter for several years to the catarrho-rheumatic ophthalmia, the last attack of which had produced penetrating ulcer of the cornea. When I saw him, all inflammation had ceased for a considerable time, there was merely a very small ulcer of the part through which the hernia protruded, attended with inability to use the eye continuously. He was on his way to Italy, to pass the winter, in order, by a residence in that milder climate, to break the habit of those regular attacks of catarrho-rheumatic ophthalmia. The hernia in the cornea was not meddled with; in fact, nothing was done, but the ulcer ultimately healed, and the keratocele disappeared, during his residence in Rome.

In the instance of Mary Falkner, the involuntary spasmodic action of the orbicularis palpebrarum muscle which existed on her entrance, was greatly alleviated by the introduction of a solution of belladonna into the eye twice daily—a beneficial effect which was even more strikingly evinced in the case of James Green. This boy æt. 9, came in the 26th of February, with scrofulous ophthalmia in both eyes, but more especially the right. He was almost unable to open this eye, from the violent spasmodic contraction of the orbicularis muscle and he was only a trifle better with regard to the left. So violent, indeed, had the action of these muscles been, that the integuments of the upper and under lids of both eyes had been brought into contact, and became extensively excoriated by their constant friction on each other, with great tendency to inversion of the lids. To remove the excoriation, a solution of sulphate of alum was employed, and that of the extract of belladonna was dropped into the eye twice a day, to relieve the spasm. The effect of the latter was most marked: in two days he could keep the eye open, unless when he spoke, and then the eye invariably became involuntarily closed, from the action of the muscle alluded to; but in two days more he was enabled to speak without this occurrence taking place, and at the end of a week he could keep his eyes open without difficulty. It may be noticed, that although the excoriation of the eyelids has disappeared, the disposition to inversion of the lids has not yet entirely so; nor will this surprise you, when we are told that this disease of the eyes has continued for a period of four years, at which time he had the small-pox.—*Lond. Med. Gaz.*

---

*On Ioduret of Iron in Hæmorrhage.* By M. Magendie.—In the course of these lectures, gentlemen, I have frequently drawn your attention to facts valuable from their direct application to the practice of our art; I have submitted the medicines in most frequent use to an experimental and physiological analysis in respect of their action on the blood, and found, sometimes to my surprise, that the results were widely different from what would generally have been expected. You saw, for example, what is likely to be the consequence of the employment of tannic acid, of Rabel water, and of citric or sulphuric lemonade, as a means of arresting hæmorrhage. You learned that all these substances liquefy the blood, and you know what that signifies. I have recently had an opportunity of putting to the test one of the results of the series of experiments to which I allude.

I had a patient affected with severe uterine hæmorrhage; my interne and myself had vainly tried all the remedies habitually used in such cases, when we thought of employing the *ioduret of iron.* You remember that that salt promotes the coagulation of blood removed from the body. A drachm of it was accordingly dissolved in two pounds of water; the patient employed it as an injection into the vagina several times in the course of the day, and on the morrow the hæmorrhage had totally ceased. I do not, however, mean to conclude from this case that the ioduret of iron must be a sovereign remedy in all similar circumstances, but I am of opinion that our success in this instance shows the propriety of having recourse to it again,—and this, for my own part, I shall not fail to do.—*Lancet.*

---

*Amputation of the Shoulder-Joint.* By Sir B. C. Brodie.—After peforming this operation on a patient who had been advised to submit to it as a last resource, Sir B. Brodie made the following "remarks" to the numerous pupils present; they embody the patient's previous history and treatment:

"This man, gentlemen, on whom you have just seen me perform this operation, was formerly in the Westminster Hospital, under the care of Mr. Guthrie, for some time, who treated the case with caustic issues, under which he improved very much, and was afterwards sent by his hospital-surgeon out of town, for the benefit of country air. He had a stiff shoulder-joint, but you know that I have often told you that a stiff joint is the best thing that can happen to a patient under such circumstances. However, this poor man could not be contented with being as well as he was, and applied to a bone-setter, who lived in the neighbourhood where he resided, and who promised to restore to him the free use of the joint again. This he did in some measure by moving the joint with very great force, and thus breaking down all the adhesions which the repeated inflammations had formed. This, as you may suppose, brought on very great and violent inflammation in the joint again; abscesses formed in the neighbourhood, and in this state the man came into the hospital. On examining the joint and the adjacent structures, I found that there were several sinuses, leading down to portions of dead bone, which I thought might exfoliate. This, however, as you have seen, was not the case, and under these circumstances two points of consideration, relative to his future treatment, presented themselves. One was to excise the joint, and the other was to amputate the limb. Mr. Babington had a case in the hospital, in which he excised the diseased joint of the shoulder; but the result was not favourable to the patient, and the man now comes backwards and forwards to the hospital, having dead pieces of bone constantly coming away. I have excised a diseased shoulder-joint in the case of a private patient, but she did not derive much benefit from it, and I have sent her into the country for change of air; but she labours also under some visceral disease, of which she will eventually die. Well, then, with these instances before me, I resolved upon performing amputation of the shoulder-joint; the poor fellow did not bear the operation so well as I could have wished, but that will not endanger his chance of eventually recovering."

*Ibid.*

